# Use of a microvascular anastomotic coupler device for kidney transplantation in rats

**DOI:** 10.1016/j.sopen.2025.01.008

**Published:** 2025-01-31

**Authors:** Henrik Lauer, Jana Ritter, Patrick Nachtnebel, Kathrin Simmendinger, Emily Lerchbaumer, Vladyslav Kavaka, Dominik Steiner, Jonas Kolbenschlag, Adrien Daigeler, Johannes C. Heinzel

**Affiliations:** Department of Hand-, Plastic, Reconstructive and Burn Surgery, BG Klinik Tuebingen, University of Tuebingen, Tuebingen, Germany

**Keywords:** Kidney transplantation, Rat model, Venous coupler device, Renal artery, Renal vein, End-to-end anastomosis

## Abstract

**Background:**

Allogenic kidney transplantation has been the gold standard treatment for end-stage renal disease. In the research setting, rat models are widely utilized to refine surgical techniques and enhance graft viability. One critical factor affecting transplantation outcomes is the efficiency of the venous anastomosis. This study evaluates the utility of a microvascular coupling device for venous anastomosis in a rat kidney transplantation model.

**Material and methods:**

Experimental allogenic kidney transplantations were conducted in male Brown Norway rats (*n* = 10) as donors and Lewis rats as recipients (*n* = 17), housed according to institutional guidelines. A microvascular coupling device was used for renal venous anastomosis, and creatinine levels were measured postoperatively to assess kidney function. Procedure times, ischemia duration, and postoperative complications were recorded and analyzed.

**Results:**

The venous anastomosis time averaged 6.6 ± 2.2 min. Total ischemia time averaged 42.4 ± 4.9 min. Early postoperative serum creatinine levels were slightly elevated about references thresholds, which normalized by postoperative day 3. Four animals died after successful transplantation due to urethral complications and postrenal failure (23.5 %). Other postoperative mortality was primarily linked to complications unrelated to thrombosis (*n* = 3, 17.6 %).

**Conclusion:**

The use of a microvascular coupling device for venous anastomosis in rat kidney transplantation significantly reduces procedure time and ischemia duration, contributing to more consistent graft outcomes. The simplification of the venous anastomosis process and reduced operative time justify the use of coupling devices. This technique holds promise for advancing preclinical transplant research and improving reproducibility in microsurgical procedures.

## Introduction

Since its first successful application in a bench-to-bedside fashion by Dr. Joseph E. Murray in 1954, allogenic kidney transplantation has become the gold standard treatment for patients with end-stage renal disease [1]. Today, rat models of renal transplantation serve as crucial platforms for advancing our understanding of surgical techniques and graft survival, aiming to improve outcomes for patients who require organ transplants [[2], [3], [4], [5]].

Following the first description of a kidney transplantation in a rat model in 1965 [6] numerous researchers have continually strived to improved surgical technique, aiming to reduce the ischemia time and thus the risk of complications during kidney transplantation in rodents [[5], [6], [7], [8]]. The success of kidney transplantation largely depends on the efficiency and precision of the anastomotic procedure, which directly influences the ischemia time and therefore overall graft viability. While anastomosis of the renal artery in rats requires microsurgical skill per se, anastomosis of the renal vein is even more demanding [5]. Although the diameter of the renal vein is significantly larger than the diameter of the renal artery, due to the thin nature of the vein wall and the ensuing risk to suture the vein's back wall during the anastomotic procedure, venous thrombosis is a feared complication in rat models of kidney transplantation [8]. Various techniques for performing venous anastomoses have been described to date, such as the use of stents or the use of cuff systems [[8], [9], [10]]. However, no “off-the-shelf” solution has yet been introduced to facilitate anastomosis of the rat renal vein.

To facilitate time-consuming, difficult venous anastomoses, the advent of so-called venous couplers has profoundly changed microsurgical tissue transfers worldwide [11,12]. While the use of venous coupling devices has been frequently reported in the literature for microsurgical applications in humans, their use in the preclinical field, particularly for anastomoses of small vessels in rodents, is sparse [13,14].

The aim of our research was to investigate the usefulness of a venous coupler system to perform microvascular anastomosis of the rat renal vein in vivo. We hypothesized that the use of such a device would prove both time-efficient and safe for renal allotransplantation in rats. We also pioneered the aforementioned technique of using a microvascular coupling device for performing venous anastomosis in a rat model of allogenic kidney transplantation for both an orthotopic as well as a heterotopic approach. It was our aim to streamline the anastomotic procedure, thereby reducing both procedural time and ischemia duration. By improving the efficiency and reliability of renal vessel anastomosis, our method purposes to contribute significantly to the field of experimental transplant surgery by improving microsurgical technique and outcomes.

## Material and methods

All animal experiments were approved beforehand by the local authorities as well as the institutional animal care and use committee (Project number: BG 02/20 G). All animals were housed and maintained according to the local and national guidelines. All procedures were carried out in full agreement with the Helsinki Declaration on Animal Rights.

### Animals

Experimental microsurgery was performed in male Brown Norway (*n* = 10) aged 8–10 weeks which served as organ donors and Lewis rats (*n* = 17) aged 12–14 weeks which served as organ recipients (Charles River, Sulzfeld, Germany). The animals' weights ranged between 225 g and 275 g. Rats were housed in groups of 5 in standard polycarbonate cages and under standardized conditions (21 °C, 12 h dark/light cycle). Water and standard rat chow were accessible ad libitum. We chose Brown Norway rats as donors and Lewis rats as recipient due to the strong RT1-mismatch between these two strains [15] which makes them a frequently and well-established model to study renal allograft rejection in rodents [16,17].

### Experimental design

All procedures were performed under an x6 up to x40 binocular operating microscope (M651 stereomicroscope, FA Leica, Wetzlar, Germany). For both donor and recipient operations, anesthesia was induced and maintained by spontaneous inhalation of isoflurane mixed with oxygen (FiO2 0.95–0.99 0.015 l/l isoflurane). For induction of anesthesia isoflurane was administered at a concentration of 5 % for 5 min, while for maintenance the concentration was lowered to 1,0-2,5 %.

Perioperative analgesia was provided by s. c. injection of buprenorphine (0.05 mg/kg), with the first dosage administered at least 60 min prior to every operation and continued every 8 h for at least the first three days after the operation or until all signs of postoperative pain had ceased. Additionally, paracetamol (3.5 mg/ml) was given orally via drinking water for the first three postoperative days. Following kidney harvest, the donor animals were sacrificed via a transthoracic administered overdose of barbiturate. The recipient animals were returned to their cages following the experimental surgeries. Given their immunogenic incompatibility, the recipient rats were treated daily with cyclosporine (10 mg/kg/d, s. c.) [18,19]. Postoperatively, rats were inspected at least once daily and checked for any signs of pain, distress or other postoperative complications. To evaluate the transplanted kidney's function, blood samples were obtained on the day of the transplantation, as well as on postoperative days 1, 3, 7 and 14. Blood samples were collected by venipuncture of the tail and care was taken to obtain no more than 1 % of the rat's estimated blood volume per day and no more than 15 % per 14 days as recommended in the literature [20]. Following the venipuncture, the blood was left to clot for 15 min before it was centrifuged at 8000 rpm for 6 min to separate the serum from the cellular components. Serum creatinine levels were then measured with an automatic analysis device (FUJI DRI-CHEM NX500, FUJIFILM Europe GmbH, Balcke-Dürr-Allee 6, 40882 Ratingen, Germany).

### Surgical technique

The rats were placed on the operating table in a supine position with abduction and external rotation of the limbs. During surgery, the animals were placed on an autoregulating warming plate and the body core temperature was maintained between 35.5 and 37.5° C. To protect the eyes against dryness, the animals' eyes were treated with an ophthalmic ointment during the surgery. The abdomen was shaved and disinfected with alcohol-soaked swabs and then covered with a sterile drape.

Experimental allogenic kidney transplantation was performed as described in the literature [10,21]. In brief, the abdominal cavity was approached through a laterally extended median laparotomy. The use of self-retractors enabled a sufficient overview of the surgical field. The bowels were covered with saline solution-soaked gauze to prevent excessive fluid loss. After moving the bowels to the right side, the left kidney was identified. The left kidney was then gently separated from the surrounding fat tissue and the left adrenal and testicular vessels with microsurgical forceps and scissors. In the donor animal, the vessels were dissected as far as possible up to the point where they joined with the abdominal aorta and inferior vena cava, respectively. In the donor rat, the adrenal and testicular vessels were clipped and subsequently transected with microsurgical scissors. In the recipient rat, those vessels were not clipped to maintain adequate blood supply to the adrenal gland and testicles. In the next step, the left ureter was exposed over a length of approximately 20 mm caudal to the renal hilum in the donor animal and 15 mm in the recipient animal. Care was taken not to separate the periureteral fat from the ureter to maintain the vascular network supplying the ureter [8].

The renal vessels were only clamped immediately before the respective transplantation in order to keep the ischemia as short as possible. In order to reduce the number of animals, the donor animal was used for two kidney recipients, i.e. both the left and the right kidney were removed and transplanted in an orthotopic and heterotopic fashion, respectively [21].

In the donor animal, the renal vessels were clamped near the aorta and the inferior vena cava in order to leave the vascular pedicle as long as possible. When the right kidney was removed, a T-shaped portion of the inferior vena cava was also excised. A clip was placed proximally to ligate the cranial aspect of the vena cava while the caudal orifice was used for venous anastomosis with the recipient vessels as described by Deng et al. [22]. The kidney was then gently flushed with low perfusion pressure using heparin solution (10 IE heparin/ml). After its removal from the donor's abdominal cavity, the kidney was immediately positioned in the recipient's abdominal cavity to avoid any cold ischemia time.

**Fig. 1:**
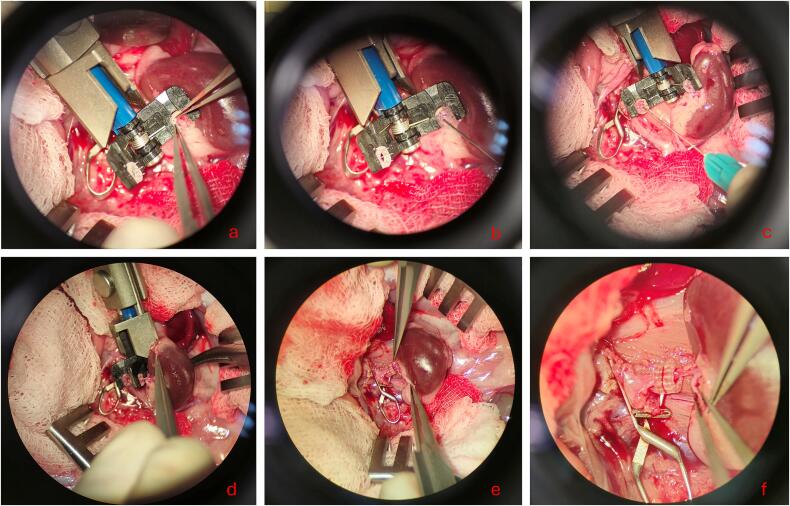
**Microvascular anastomosis of the rat renal vein. a:** The donor renal vein is gently pulled through one of the coupler rings. **b:** The donor renal vein is flushed with heparin solution **c:** The recipient renal vein is pulled through the other coupler ring and also gently flushed with heparin solution. **d:** The coupler rings are approximated via the coupling device. **e:** The coupling device is removed. **f:** Completion of the venous anastomosis.

Microvascular anastomosis of the renal vein was performed before the respective arterial anastomosis. For this purpose, a microvascular coupling device (1,0–1,5 mm, GEM COUPLER, FA Synovis Micro Companies Alliance, Birmingham, USA) was used (Fig. 1a-f).

First, the donor renal vein was gently pulled through one of the coupler ring's orifices with blunt microsurgical forceps (Fig. 1a) and flushed with heparin solution (Fig. 1b). Under careful control of the kidney to avoid any tension on the renal vessels, the coupler device was then repositioned, and the recipient renal vein was pulled through the other coupler ring in similar fashion (Fig. 1c). The coupler rings were then approximated by means of gentle rotational movements of the coupling device while utmost care was taken to avoid any tension on the renal vessels (Fig. 1d). Finally, after both rings were carefully approximated, final contact was established by means of gentle pressure with an atraumatic vessel clamp (Fig. 1e). With the coupler rings now closed the coupler device was removed from the surgical field and the microvascular anastomosis of the renal vein was completed (Fig. 1f).

**Fig. 2:**
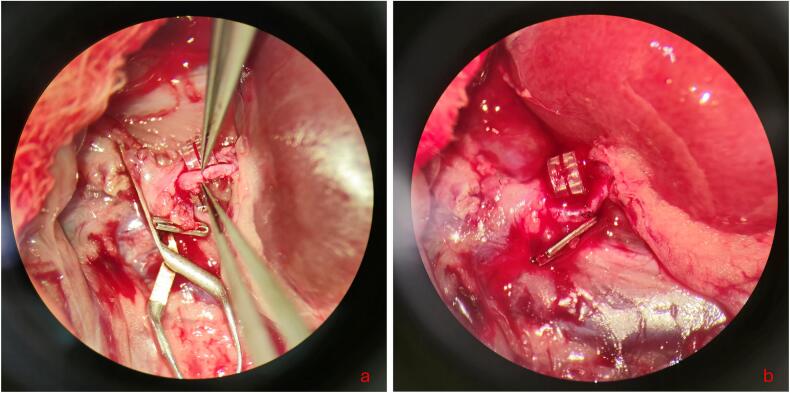
**Microvascular anastomosis of the rat renal artery. a:** The arterial vessels are gently prepared and any protruding adventitia is removed. Note that the coupler ring serves as an abutment for any manipulation of the renal artery. **b**: End of the anastomosis procedure. Once the vessels clamps have been released, reperfusion of the kidney becomes evident by a marked color change from grey to red. (For interpretation of the references to color in this figure legend, the reader is referred to the web version of this article.)

The arterial anastomosis (Fig. 2a-b) was then performed in end-to-end technique with simple interrupted 10–0 sutures (Ethilon, FA Ethicon, Johnson and Johnson, New Brunswick, USA). The coupler rings proved hereby helpful as an abutment (Fig. 2a). We recommend performing corner sutures first and then closing the front and back wall using interrupted sutures. This avoids the risk of stitching the opposite wall during the anastomosis. Following the completion of both the venous and arterial anastomosis, the vessels clamps were released with outmost care to avoid any damage to the renal artery and vein. In case of persistent bleeding from the anastomosis, the anastomosis site was gently compressed with sterile cotton swabs for approximately 30 s until no more bleeding was visible. Reperfusion of the kidney became quickly apparent within the first three minutes after release of the vessels clamps as indicated by a distinct color change from grey to red (Fig. 2b).

The ureteral anastomosis was performed after the microvascular anastomoses and as soon as reperfusion of the transplanted kidney was secured. After identification of the lumen this step of the surgery was performed in the same way as the arterial anastomosis using 10–0 simple interrupted sutures.

In case of an orthotopic transplantation, i.e. implantation of the donor's right kidney in the original situs of the recipient's left kidney, the same approach was used but the donor kidney was rotated by 180° to enable topographical correct anastomoses of the renal vessels and ureter, respectively.

Following the completion of the kidney transplantation, the recipient's right kidney was approached in an analogous fashion and the right ureter was gently freed from the surrounding fat and ligated with a microvascular clip. Next, the right kidney was carefully freed from the surrounding tissue with outmost care not to damage the suprarenal gland or vessels, respectively. Finally, the renal vein and artery were dissected and ligated with microvascular clamps before the nephrectomy was completed.

After the abdominal cavity was carefully rinsed with sterile saline solution, the abdominal wall and skin were closed in layers using absorbable 5–0 vicryl sutures.

### Statistical analysis

Statistical analysis was performed with IBM SPSS Statistics 29.0.

## Results

Choice of the size of the microvascular anastomotic coupler device.

In the majority of rats, a 1.5 mm coupler device was used for venous anastomosis. Only in one animal a 1.0 mm coupler was chosen due to the smaller diameter of the original renal vein.

### Animal welfare and perioperative complications

Four animals (23.5 %) were sacrificed prior to the third postoperative days due to progressive apathy and poor overall physical condition. One animal died due to an isoflurane overdose while under anesthesia while another one was found dead in its home cage approximately 6 h after the surgery had been successfully completed. One recipient suffered from extensive blood loss during the surgery due to a leaky venous anastomosis and had to be sacrificed on the third postoperative day because of its poor overall physical condition. In all cases autopsy was performed immediately after the animals had been sacrificed to determine the underlying reason for the animals' poor condition. These autopsies showed no evidence of thrombosis of the renal vessels or ischemia or necrosis of the kidney. However, the ureter was found to be non-patent in the area of the anastomosis and appeared massively dilated proximally to the anastomosis site, revealing postrenal failure as underlying reason (*n* = 4; 23.5 %).

### Postoperative serum creatinine levels

**Fig. 3:**
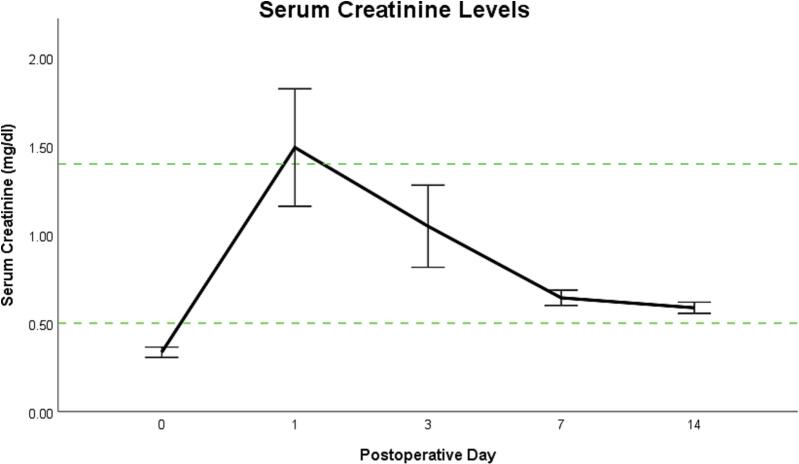
**Average serum creatinine levels before and after allogenic kidney transplantation.** The green lines indicate the respective upper (1.40 mg/dl) and lower (0.50 mg/dl) reference thresholds for serum creatine in rats as set by the automatic analysis device used in this study. Note from postoperative day 3 onward, all serum creatine levels were within normal reference ranges. (For interpretation of the references to color in this figure legend, the reader is referred to the web version of this article.)

Preoperatively, the average serum creatinine was measured at 0.33 (±0.07) mg/dl. On the first operative day, serum creatinine levels increased to 1.49 (±1.05) mg/dl, which was slightly above the upper reference threshold of 1.4 mg/dl. On the third postoperative day, serum creatinine levels decreased to 1.04 (±0.73) mg/dl. Seven and fourteen days postoperatively, serum creatinine levels of 0.64 (±0.13) and 0.58 (±0.09) respectively were measured, which was within normal reference values on both occasions (Fig. 3).

### Required time for venous and arterial anastomosis as well as total ischemia time

**Fig. 4:**
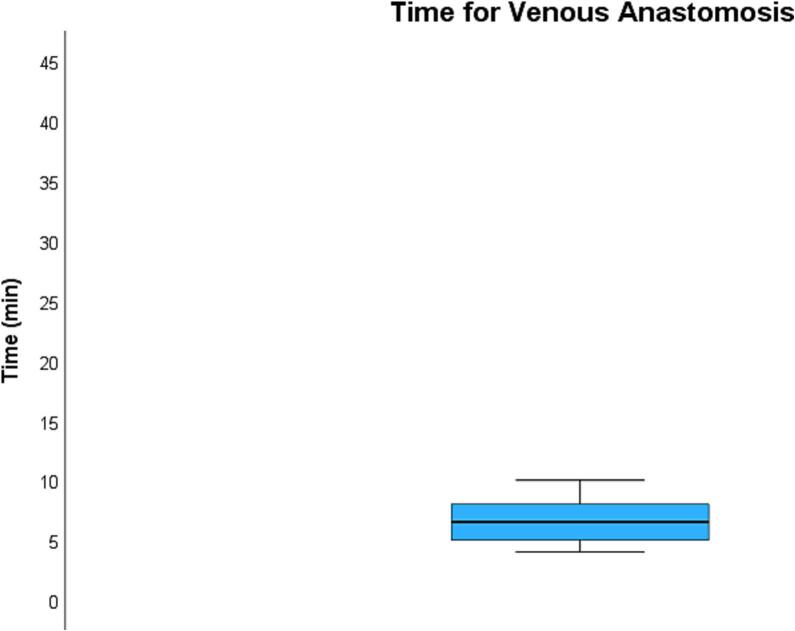
**Average time required for venous anastomosis.** The average time required to perform the venous anastomosis was 6.6 ± 2.2 min.

**Fig. 5:**
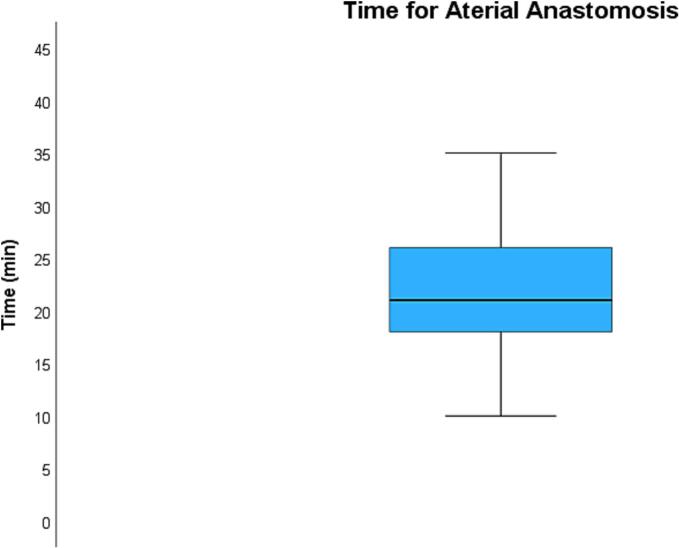
**Average time required for arterial anastomosis.** The average time required to perform the arterial anastomosis was 21.6 ± 6.8 min.

**Fig. 6:**
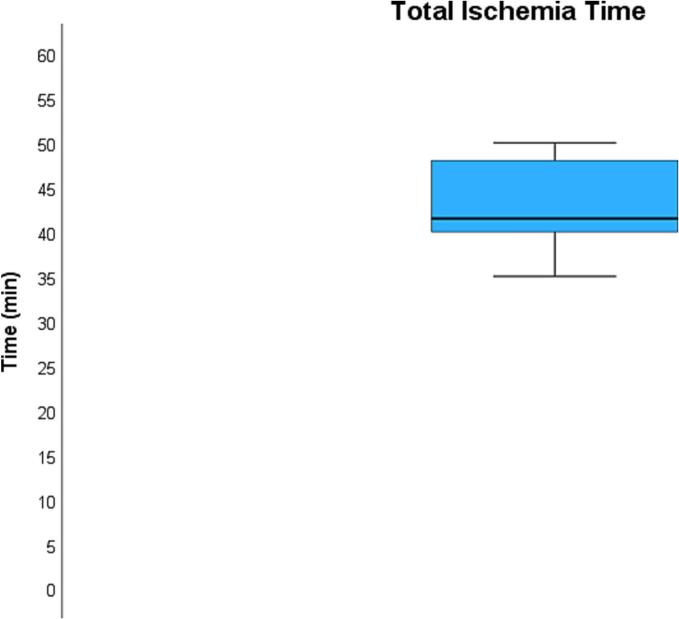
**Average total ischemia time.** Total kidney ischemia time averaged at 42.4 ± 4.9 min.

The average time required to perform the venous anastomosis (Fig. 4) was 6.6 ± 2.2 min while the average time required to perform the arterial anastomosis (Fig. 5) was 21.6 ± 6.8 min. Total kidney ischemia time (Fig. 6) averaged at 42.4 ± 4.9 min.

## Discussion

In this study, we present a novel surgical technique for renal venous anastomosis in a rat model of allogenic kidney transplantation by using a microvascular anastomotic device. We aimed to investigate whether the use of this technique could reduce the time required for the anastomotic procedure, therefore minimizing ischemia times during transplantation. The use of microvascular coupling devices is characterized by simplicity and precision, which are critical factors in enhancing surgical outcomes and reducing potential complications [11,23].

Performing a venous anastomosis is considered to be far more technically demanding than arterial anastomosis, especially in preclinical models [24]. For this reason, previous studies have investigated various methods for renal anastomosis, each with its own advantages and limitations as follows: Traditional hand-sewn end-to-end techniques for venous anastomosis often require meticulous vessel dissection and suturing, which can prolong ischemia time and increase the risk of technical errors [[25], [26], [27]]. Other approaches, such as the use of cuffs or sleeves have been explored to streamline the process; however, these methods may introduce additional complexity or require specialized training [8,26,28]. Additionally, neither cuffs nor sleeves are available in an off-the-shelf- fashion as they have to been handcrafted for each procedure in accordance with specific requirements in regard to the diameter of the respective vessels.

Since its introduction in 1962, the technique of mechanical coupler connection has been modified and used in the specialist disciplines of otorhinolaryngology, maxillofacial surgery and plastic surgery [[29], [30], [31], [32]]. Even if the use of a coupler device appears more expensive compared to conventional manual suturing, it has been shown that the costs are amortized in the clinical situation through repeated applications due to the cumulatively shorter operating time and the lower complication rate [13,33].

In our study, the time required for venous anastomosis averaged at 6.6 min, a rather short duration for this critical step of the operation when compared to the results published in the literature. When reviewing the various published anastomosis techniques, we found comparable venous anastomosis times only in the study by Jin et al. with an average of 9.2 min, which used a stent-sleeve technique [8]. Therefore, our technique poses an almost 30 % reduction in anastomosis time, which can be considered crucial as prolonged ischemia can have detrimental effects on graft viability and function [34]. Other studies on rat kidney transplantation have reported significantly longer times for venous anastomosis, e.g. 19.30 min in case the venous anastomosis was performed by a combination of end-to-end sutures with an epidural catheter [35]. Deng et al. compared four different techniques for venous anastomosis of the rat kidney. The reported venous anastomosis times of 18.45 min for the modified end-to-end group, 17.95 min for the end-to-side group, 32.60 min in case of a vena-cava-bypass and 17.60 min for the conventional end-to-end technique [22]. These results emphasize the time saving achievable by using a microvascular coupling device. Several studies have recommended that overall ischemia time of the allogenic organ transplant should not exceed 45 min [10,36,37]. In our study we were able to show that this threshold was achievable with an average ischemia time of less than 43 min when using a microvascular anastomotic device. While we were able to prove that the use of a microvascular anastomotic device results in both a reduced duration to perform the venous anastomosis as well as overall ischemia time, the material costs associated with the use of such a device are substantially higher in comparison to other published techniques. While the price for one microvascular anastomotic device ranges around 300€ [38] material costs both for suture material for microvascular procedures as well as the aforementioned cuffs or sleeves are substantially lower.

Nevertheless, use of a microvascular coupler device offers several advantages in our opinion which outweigh the system's higher cost. One the one hand, we see a clear advantage in the simplification of the venous anastomosis, especially when performing an orthotopic transplantation of the right kidney which is aggravated due to the short length of the original vessels. This aspect is particularly advantageous as it expands the pool of available organs for transplantation, thereby enhancing research opportunities and potential clinical applications, which is also in line with the “3R” of animal research [21,39,40].

On the other hand, the use of an off-the-shelf product mitigates several problems associated with the use of custom-made cuff or stent-systems. While the latter approaches require a case-based adjustment to fit specific requirements in regard to the original vessel's diameter, the use of microvascular coupler rings, which can be purchased in different sizes, spares the surgeon a time-consuming but crucial aspect of the operation.

Last but not least, the microvascular anastomotic device can serve as an abutment during surgery while performing the arterial anastomosis, facilitating the quick and efficient completion of this crucial step of transplantation surgery. Although a recent survey revealed that surgeons are generally reluctant to use microvascular coupling devices for the arterial anastomosis we would deem it interesting to investigate whether the arterial anastomosis can be performed faster when such a device was used to for experimental renal transplantation when compared to traditional, i.e. hand-sewn microvascular anastomosis [41].

Regarding the survival rate and complication reported in this work, only 59.8 % of recipients survived until postoperative day 14. This rate is substantially lower than rates reported by other authors, e.g. Karatzas et al. achieved a survival rate of 87 % until the 14th postoperative day [7]. Interestingly, the aforementioned authors performed the right nephrectomy one week postoperatively, contrasting our approach which includes simultaneous right nephrectomy at the time of transplantation surgery. Noteworthy, simultaneous bilateral nephrectomy is associated with mortality rates of up to 70 % whereas this rate is significantly lower following 2-stage nephrectomy [42]. In our study however, a 2-stage nephrectomy was not feasible due to the experimental design. It must be noted that the complications which occurred in our study were not related to vascular problems, i.e. arterial or venous thrombosis but mainly to urinary complications, i.e. stenosis of the ureter, which occurred in 4/7 animals. Since we used the technically more difficult end-to-end-technique for the ureteral anastomosis, an overall urinary complication rate of 23.5 % (4/17 animals) can be considered within normal ranges as rates of up to 80 % have been reported in the literature [25,42]. Although the use of stents is considered risky by some authors [25] recent studies reported low complication rates when using this technique [10]. The “bladder insertion” technique as reported by Yin [43] and Schumacher [27] has been suggested as promising alternative to avoid urinary complications [25,42].

### Limitation

The rat kidney transplantation model is a microsurgical challenge and is associated with a steep learning curve. Even though our results show clear advantages regarding operation times when a microvascular anastomotic device is used, this technique is very difficult to perform by a single surgeon, a significant limitation which must be considered when planning and performing experiments using this technique. The high costs for microvascular coupling devices are also a noteworthy limitation but in our opinion are outweighed by this technique's feasibility and success rate.

## Conclusion

The venous coupler approach to renal anastomosis in rat kidney transplantation demonstrates improvements in comparison to traditional techniques. By reducing both procedural time and ischemia duration while ensuring ease of execution, this method holds promise for improving outcomes in experimental kidney transplantation.

## CRediT authorship contribution statement

**Henrik Lauer:** Writing – original draft, Visualization, Resources, Project administration, Investigation, Data curation, Conceptualization. **Jana Ritter:** Writing – review & editing, Resources, Investigation, Data curation. **Patrick Nachtnebel:** Investigation. **Kathrin Simmendinger:** Investigation. **Emily Lerchbaumer:** Writing – review & editing, Visualization. **Vladyslav Kavaka:** Writing – review & editing, Formal analysis. **Dominik Steiner:** Writing – review & editing, Validation. **Jonas Kolbenschlag:** Writing – review & editing, Supervision, Resources, Methodology, Funding acquisition. **Adrien Daigeler:** Writing – review & editing, Supervision, Funding acquisition. **Johannes C. Heinzel:** Writing – original draft, Validation, Software, Resources, Project administration, Methodology, Investigation, Formal analysis, Data curation.

## Ethical approval

All procedures were performed in accordance with the international animal care regulations and approved by the regional authorities.

## Funding

The authors received no financial support for the preparation, research, authorship, and/or publication of this manuscript. We acknowledge support by Open Access Publication Fund by University of Tuebingen.

## Declaration of competing interest

The microvascular anastomotic coupler devices used in this study were provided free of charge by TapMed Medizintechnik Handels GmbH, Gewerbepark 10, 34317 Habichtswald-Ehlen, Germany. The authors have no financial relationship with TapMed in any kind.

JH received free food from Axogen® at several locations. JK works as a consultant for Axogen®.
